# Index of Volume One

**Published:** 1918-12

**Authors:** 


### INDEX OF VOLUME ONE

As many medical officers have expressed the desire to preserve the numbers of *The Medical Bulletin* and *War Medicine* in bound form, an index for Volume I has been prepared for distribution with the current number of *War Medicine*. This index covers the numbers from November 1917 through June-July, 1918, all but the last of which appeared under the title *The Medical Bulletin*. Another index covering the remaining numbers is in preparation, and will be distributed when Volume II is complete.

